# Bimodal Absorber Frequencies Shift Induced by the Coupling of Bright and Dark Modes

**DOI:** 10.3390/ma17133379

**Published:** 2024-07-08

**Authors:** Yun Chen, Jiangbo Hu, Shan Yin, Wentao Zhang, Wei Huang

**Affiliations:** 1 Guangxi Key Laboratory of Optoelectronic Information Processing, School of Optoelectronic Engineering, Guilin University of Electronic Technology, Guilin 541004, China; yun_chen2022@163.com (Y.C.); syin@guet.edu.cn (S.Y.); 2 School of Physical Science and Technology, Guangxi Normal University, Guilin 541004, China; 3 Institute of Scientific and Technical Information of Guangxi Zhuang Autonomous Region, Nanning 530022, China; 18577786166@163.com

**Keywords:** terahertz, metamaterial, coupled-mode theory, interference theory, absorber

## Abstract

In this paper, we demonstrate that the absorption frequencies of the bimodal absorber shift with the coupling strength of the bright and dark modes. The coupling between the bright mode and the dark mode can acquire electromagnetically induced transparency, we obtain the analytical relationship between the absorbing frequencies, the resonant frequencies, losses of the bright mode and dark mode, and the coupling strength between two modes by combining the coupled mode theory with the interference theory. As the coupling strength between the bright mode and the dark mode decreases, the two absorption peaks gradually move closer to each other, inversely, they will move away from each other. The simulation employs three distinct metasurface structures with coupling of the bright and dark modes, thereby verifying the generality of the theoretical findings.

## 1. Introduction

Terahertz waves have extensive applications in security detection [1], biosensing [2,3], and wireless communication due to their superior penetration capability and low energy (compared to infrared light) [4]. Metamaterials are a class of artificial composite materials with special physical properties that cannot be achieved by natural materials. They have promising prospects for electromagnetic stealth, sensing, antenna design, wavefront control, and other fields [5,6]. The thickness of metasurfaces is much smaller than the wavelength and can be considered two-dimensional metamaterials [7]. Through the artificial design, a metasurface structure with arbitrary permittivity and permeability can be realized to modulation the amplitude, phase, and polarization of the THz beam [8,9].

Coupled mode theory (CMT) is a universal theory to describe the coupling of different electromagnetic modes. It was originally used to describe the coupling of waveguide modes [10], and then introduced into metasurface systems to explain the physical mechanism of Fano resonance [11,12,13], bound states in the continuum (BIC) [14,15,16,17,18,19], electromagnetically induced transparency and other optical phenomena [20,21]. The couple-mode theory is a fundamental theory to explain the generation of the EIT [21], and the basic physical parameters in the couple-mode equation are closely related to the geometric parameters of the metasurface structures, thus the EIT response can be tunable by varying the structural parameters. The modes that can be directly stimulated by the incident electromagnetic waves are called bright modes, and the modes that cannot be directly stimulated by the incident electromagnetic waves are called dark modes [20]. When it comes to the coupling between bright and dark modes in metasurfaces, the most typical example is electromagnetically induced transparency (EIT) [22]. EIT is a quantum interference effect that occurs in a three-level atomic system and produces sharp transparent windows within a wide transmission spectrum [23]. Although EIT was first proposed in quantum mechanics [24,25,26], it has been extensively studied in terahertz metasurface in recent years [27,28]. EIT effect significantly changes the dispersion characteristics of metasurface structure, which leads to the light slowing phenomenon and nonlinear enhancement effect [29,30,31,32]. It has extensive applications such as optical storage [33], sensors [34], frequency conversion [35,36], chiral [37] and other fields.

The wave absorbing stealth technology is a type of metamaterial stealth technology. Typically, the metamaterial absorbers consist of a metal-dielectric-metal (MDM) sandwiched structure, comprising a periodic-patterned metal layer, a dielectric spacer layer, and a metal plane. The absorption mechanism of the absorber is commonly explained using optical modulator theory, impedance matching theory [38,39], interference theory [40,41], and critical coupling theory [42,43]. By optimizing the structural parameters, the reflection and transmission at a certain frequency are minimized simultaneously thus achieving the perfect absorption at this frequency. Recently, there have been studies utilizing VO_2_ phase transition material as the substrate material for EIT metamaterials [44,45]. By changing the environmental temperature, the substrate material undergoes a phase transition to either dielectric or metallic states, enabling switching from transmitted EIT metamaterials to absorbing metamaterials [46]. Despite previous research on EIT-to-absorber conversion, the analytical relationship between the coupling of the bright and dark modes and the absorption frequency has not been investigated.

The motivations and novelties behind this paper are as follows: Firstly, we establish the connection between the transmission and reflection spectra obtained from the coupled-mode theory and the absorption spectra described by the interference theory by combining the two theories. Moreover, we propose a novel concept for the inverse design of absorber metamaterials. The theoretical findings presented in this study can be applied to the inverse design process of absorber metamaterials. In step one, by considering the desired absorption frequency, the resonance frequency and loss of the structure can be determined; subsequently, based on the relationship between resonance frequency, loss, and structural geometric parameters, the fundamental size of the structure can be determined. At this stage, there may exist slight deviations between absorption frequency and expected value; therefore, further adjustments are made by modifying the coupling among structures to achieve more accurate results. Furthermore, we provide a systematic analysis of the influence of bright mode and dark mode coupling on the shift in absorption frequency. The analytical results of the absorption frequency are obtained through theoretical derivation and analyze the shift of the absorption frequencies with varies in coupling between the bright mode and dark mode by combining simulation results. The two absorption peaks gradually approach each other as the coupling between the structures weakens. When the coupling between the bright and the dark modes decreases to a certain value, the dark mode will no longer be excited by the bright mode. As a result, the two absorption peaks will gradually merge into one, which is essentially consistent with the absorption spectrum observed when only the bright mode is present. Nevertheless, the proposed method exhibits universality in coupling between bright and dark modes in metal metamaterials.

In this paper, we demonstrate the coupling of bright mode and dark mode by employing different typical metasurfaces that can acquire EIT. We employ the metal cut wires (CWs) as the bright mode structures, and three different metasurface structures, U-shape, split ring resonances (SRRs), and C-shape split ring resonances (CSRRs) are used as the dark mode structures. We transform EIT into an MDM absorber by adding a metal reflective layer on the back of the substrate and analyze the relationship between the absorption frequency and the basic physical parameters. We obtain the relationship between the absorber frequencies, the coupling strength between bright mode and dark mode, and the frequencies and the losses of the two modes by combining the coupled-mode theory and interference theory. Due to the basic physical parameters in the coupled-mode theory being closely related to the geometric parameters of the structure, the inverse design of the metamaterial structure parameters can be acquired by employing the relationship between absorption frequency and the physical parameters (such as resonance frequency, loss, and coupling strength) in the coupled-mode theory.

## 2. Theory and Methods

Analogous to the electromagnetically induced transparency (EIT) phenomenon in a three-level quantum system, the resonance of the bright and dark modes in the metasurface corresponds to two excited states $|1\rangle$ and $|2\rangle$. Both modes are in the ground state $|0\rangle$ when they are not excited, the bright mode can be directly excited by the incident light ($|0\rangle$→$|1\rangle$), and the dark mode cannot be directly excited by the incident light, but can be excited by the bright mode through the coupling between them ($|1\rangle$→$|2\rangle$), thus, the destructive interference between pathways $|0\rangle$→$|1\rangle$ and $|0\rangle$→$|1\rangle$→$|2\rangle$→$|1\rangle$ is formed. The EIT produces strong loss suppression and large dispersion in a narrow band, resulting in a large group delay which leads to the slow light effect.

Based on the coupled mode theory, if both modes can be directly excited by the incident electromagnetic wave simultaneously, then the coupling equation can be written as follows [15],

(1) $$\left[\begin{array}{cc} \omega -\omega_{a}-i\gamma_{a} & \;g\\ \;g & \omega -\omega_{b}-i\gamma_{b} \end{array}\right]\left[\begin{array}{c} a\\ b \end{array}\right]=\left[\begin{array}{c} \sqrt{\gamma_{a}}E\\ \sqrt{\gamma_{b}}E \end{array}\right],$$

where ω represents the frequency of the incident terahertz wave. $\omega_{a}$ and $\omega_{b}$ represent the resonance frequencies of the two modes. We take the *a* and *b* as the energy amplitudes of each structure in the unit cell. $\gamma_{a}$ and $\gamma_{b}$ are the loss terms of two modes. *g* represents the coupling strength between two modes. *E* is the energy amplitude of the externally excited terahertz wave.

The coupled-mode theory can also describe the coupling between bright mode (CW) and dark mode (U-shape, SRRs, and CSRRs) to acquire electromagnetically induced transparency due to the coupling of the electromagnetic modes. In this case, only the bright mode can be directly excited by the incident electromagnetic wave, so the excitation term of the bright mode on the right side of the coupled mode equation (Equation (2)) is written as $\sqrt{\gamma_{1}}E$, while the dark mode cannot be directly excited by the incident light and the excitation term is written as zero. We take the ${\left|a\right|}^{2}$ and ${\left|b\right|}^{2}$ as the energies of each structure in the unit cell. Thus, the coupled equations for the bright and dark modes can be written as follows [27],

(2) $$\left[\begin{array}{cc} \omega -\omega_{1}-i\gamma_{1} & \;g\\ \;g & \omega -\omega_{2}-i\gamma_{2} \end{array}\right]\left[\begin{array}{c} a\\ b \end{array}\right]=\left[\begin{array}{c} \sqrt{\gamma_{1}}E\\ 0 \end{array}\right],$$

where $\omega_{1}$ and $\omega_{2}$ represent the resonance frequencies of the bright mode and dark mode under different polarization states (TE/TM), respectively. $\gamma_{1}$ and $\gamma_{2}$ are the loss terms of two modes. The remaining parameters are defined as stated in Equation (1).

By solving the coupled mode equation, we obtain the expressions for the energy amplitudes *a*, and *b* of each mode as follows.

(3) $$a=\frac{-\sqrt{\gamma_{1}}\left(\omega -\omega_{2}-i\gamma_{2}\right)E}{\;g^{2}-\left(\omega -\omega_{1}-i\gamma_{1}\right)\left(\omega -\omega_{2}-i\gamma_{2}\right)};$$

(4) $$b=\frac{g\sqrt{\gamma_{1}}E}{\;g^{2}-\left(\omega -\omega_{1}-i\gamma_{1}\right)\left(\omega -\omega_{2}-i\gamma_{2}\right)}.$$

The effective electric susceptibility is the linear superposition of the energy amplitudes ${\left|a\right|}^{2}$ and ${\left|b\right|}^{2}$, which can be written as [15]

(5) $$\chi_{\mathrm{eff}}\approx \frac{\sqrt{\gamma_{1}}a+0\cdot b}{\varepsilon_{0}E},$$

Since dark mode cannot be directly excited by the external electromagnetic field, therefore, the excitation term of the dark mode is zero in Equation (5). $\varepsilon_{0}$ is the relative permittivity of the background. Finally, we obtain the transmission spectrum with the expression $T\approx 1-Im(\chi_{\mathrm{eff}})$ [11], as follows Equation (6). We can also obtain the approximate expression of the reflective spectrum as follows Equation (7).

(6) $$T\approx 1-Im\left(\frac{-\gamma_{1}\left(\omega -\omega_{2}-i\gamma_{2}\right)}{\;g^{2}-\left(\omega -\omega_{1}-i\gamma_{1}\right)\left(\omega -\omega_{2}-i\gamma_{2}\right)}\right).$$

(7) $$R\approx Im\left(\frac{-\gamma_{1}\left(\omega -\omega_{2}-i\gamma_{2}\right)}{\;g^{2}-\left(\omega -\omega_{1}-i\gamma_{1}\right)\left(\omega -\omega_{2}-i\gamma_{2}\right)}\right).$$

Due to the dark mode cannot be excited directly by the incident electromagnetic field, but by the electromagnetic field of the bright mode, we assume that the resonance frequencies of the bright mode and the dark mode are approximately equal, that is, $\omega_{1}\approx \omega_{2}\approx \omega_{0}$. By simplifying Equations (6) and (7), the expressions of the transmission and reflective spectra can be written as follows.

(8) $$T\approx 1-\frac{\gamma_{1}\gamma_{2}\left[g^{2}-{\left(\omega -\omega_{0}\right)}^{2}+\gamma_{1}\gamma_{2}\right]+\gamma_{1}{\left(\omega -\omega_{0}\right)}^{2}\left(\gamma_{1}+\gamma_{2}\right)}{{\left[g^{2}-{\left(\omega -\omega_{0}\right)}^{2}+\gamma_{1}\gamma_{2}\right]}^{2}+{\left[\left(\omega -\omega_{0}\right)\left(\gamma_{1}+\gamma_{2}\right)\right]}^{2}}.$$

(9) $$R\approx \frac{\gamma_{1}\gamma_{2}\left[g^{2}-{\left(\omega -\omega_{0}\right)}^{2}+\gamma_{1}\gamma_{2}\right]+\gamma_{1}{\left(\omega -\omega_{0}\right)}^{2}\left(\gamma_{1}+\gamma_{2}\right)}{{\left[g^{2}-{\left(\omega -\omega_{0}\right)}^{2}+\gamma_{1}\gamma_{2}\right]}^{2}+{\left[\left(\omega -\omega_{0}\right)\left(\gamma_{1}+\gamma_{2}\right)\right]}^{2}}.$$

When we add a metal reflecting film to the back of the substrate, the original EIT metasurface becomes a typical MDM absorber. The physical mechanism of absorption can be explained using interference theory, which is expressed as follows [41],

(10) $$r_{total}=r_{11}+\frac{t_{12}t_{21}r_{23}e^{i2\beta }}{1-r_{22}r_{23}e^{i2\beta }},$$

where $r_{total}$ represents the total reflection coefficient of the metasurface, $r_{11}$ represents the reflection coefficient between the metasurface and air, the $r_{22}$ represents the reflection coefficient between the metasurface and dielectric layer, and the $r_{23}$ represents the reflection coefficient between the metal film and dielectric layer. The metal film can prevent the incident electromagnetic wave from passing through, and act as a mirror, we assume that the direction of the incoming electromagnetic wave is positive, so $r_{23}=-1$. $t_{12}$ is the transmission coefficient from the metasurface to the dielectric layer, and $t_{21}$ is the transmission coefficient from the dielectric layer to the metasurface. In this paper, only the case of normal incidence is considered. $\beta =\sqrt{\varepsilon_{spacer}}k_{0}d$ is the propagation phase, $\varepsilon_{spacer}$ is the relative permittivity of spacer layer, $k_{0}$ is the wavenumber of the free space, *d* is the thickness of the dielectric layer. Due to the thickness of the dielectric layer being much smaller than the incident wavelength, the perturbation introduced by the propagation phase is neglected. Since there is a metal film on the back of the substrate, it can be considered that no light can be transmitted, and according to the formula of absorption $A=1-{\left|T\right|}^{2}-{\left|R\right|}^{2}$ [41], the absorption can reach a maximum value when the total reflection is minimum. We find that the transmission and reflection coefficients of the metasurface obtained by the coupled mode theory and the interference theory are consistent, that is $R=r_{11}=r_{22}$, $T=t_{12}=t_{21}$.

Then substituting Equations (8) and (9) into Equation (10), we can obtain the expression of the total reflection coefficient ($r_{total}$) as follows,

(11) $$r_{total}\approx \frac{4\gamma_{1}\gamma_{2}\left[g^{2}-{\left(\omega -\omega_{0}\right)}^{2}+\gamma_{1}\gamma_{2}\right]+4\gamma_{1}{\left(\omega -\omega_{0}\right)}^{2}\left(\gamma_{1}+\gamma_{2}\right)}{{\left[g^{2}-{\left(\omega -\omega_{0}\right)}^{2}+\gamma_{1}\gamma_{2}\right]}^{2}+{\left[\left(\omega -\omega_{0}\right)\left(\gamma_{1}+\gamma_{2}\right)\right]}^{2}+\gamma_{1}\gamma_{2}\left[g^{2}-{\left(\omega -\omega_{0}\right)}^{2}+\gamma_{1}\gamma_{2}\right]+\gamma_{1}{\left(\omega -\omega_{0}\right)}^{2}\left(\gamma_{1}+\gamma_{2}\right)}-1.$$

Therefore, we solve Equation (11) under the condition that the total reflection ($r_{total}$) is minimized, we can obtain the expression as Equation (12).

Ignoring the higher-order terms in the formula, namely, $\gamma_{1}^{2}\gamma_{2}^{2}=0$, $\gamma_{1}\gamma_{2}g^{2}=0$, $g^{4}=0$, then,

(12) $$2\gamma_{1}^{2}{\left(\omega -\omega_{0}\right)}^{2}-\gamma_{2}^{2}{\left(\omega -\omega_{0}\right)}^{2}={\left(\omega -\omega_{0}\right)}^{4}-2g^{2}{\left(\omega -\omega_{0}\right)}^{2}.$$

Furthermore, we combine Equation (2) to Equation (12), thus obtaining the relationship between the absorption frequencies, resonant frequencies, and losses of the bright mode and dark mode, the coupling strength between the two modes is as follows,

(13) $$\omega_{\pm }=\omega_{0}\pm \sqrt{2g^{2}+2\gamma_{1}^{2}-\gamma_{2}^{2}},$$

where $\omega_{+}$ represents the higher absorption frequency and $\omega_{-}$ represents the lower absorption frequency. *g* represents the coupling strength between the bright and dark modes, $\gamma_{1}$, and $\gamma_{2}$ represent the losses of bright mode and dark mode, respectively.

The physical parameters in the coupled-mode theory are obtained from the basic physical parameters of the two structures, such as the resonant frequency $\omega_{1}$, $\omega_{2}$, the losses $\gamma_{1}$, $\gamma_{2}$ and the coupling strength *g* between the structures in one unit cell. Therefore, when the structure parameters of the EIT are given, the location of the absorption peak frequencies can be predicted if we know the coupling strength between the two structures in the unit cell.

Although coupled-mode theory and interference theory describe distinct physical processes, the combination of two theories establishes a connection between the transmission and reflection spectra obtained from the coupled-mode theory and the absorption spectra described by the interference theory. Theoretical derivation enables us to establish relationships between absorption frequency, resonance frequency, loss, and coupling strength of the structure. As basic physical parameters in the coupled-mode theory are closely related to geometric parameters of the structure, inverse design of metamaterial structure parameters can be achieved by utilizing the relationship between absorption frequency and physical parameters (such as resonance frequency, loss, and coupling strength) in the coupled-mode theory.

## 3. Results

We use a frequency-domain solver in the CST Microwave Studio to obtain the transmission spectra of the EIT metasurface structures in simulations. The boundary conditions in the *x* and *y* directions are set to the unit cell. The open (add space) boundary condition is set in $Z_{max}$ (incidence direction), and the open and open (add space) boundary conditions are set in $Z_{min}$ (exit direction) corresponding to transmission and absorption metamaterial, respectively. The number of modes used in the simulation is 60. For the bright modes, the unexcited dark modes, and the whole structures, the polarization direction of the incident electromagnetic wave is the *y* polarization, and the polarization direction of the excited dark mode is the *x* polarization. The meshes we use in simulations are tetrahedral, and the meshes are refined for more accurate results. The mesh settings for the model and background are 10 cells and 1 cell per max model box edge, respectively.

The material of the spacer layer is silicon with a relative permittivity of 11.7 [19], and the metal structure and the metal plane are all aluminum. The conductivity of aluminum is set to $3.56\times 10^{7}$ S/m in the simulation. The thickness of the dielectric and metal layers are 50 μm and 200 nm, respectively. We optimize the structural parameters to make the absorber achieve perfect absorption close to unity as possible.

**Figure 1:**
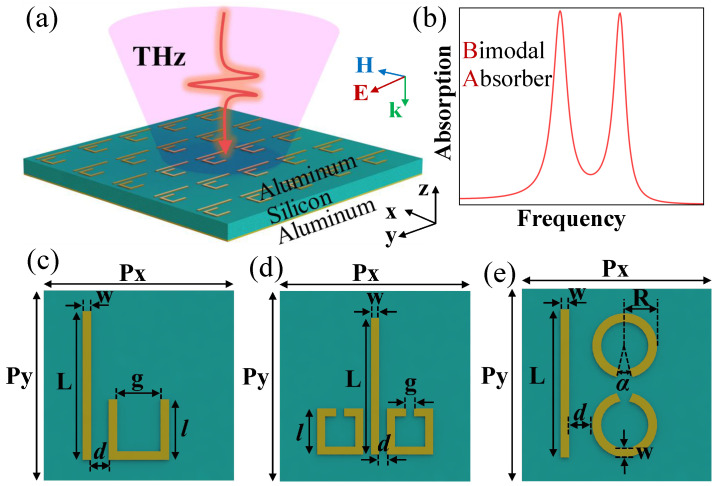
(**a**) The schematic figure of THz bimodal absorber. (**b**) The schematic figure of the absorption spectrum. (**c**–**e**) are examples of the different unit cells of metamaterial devices with different structures of bright modes and dark modes. The geometrical parameters of (**c**) are $P_{x}=P_{y}=110\;\;\mu$m, L=86 μm, l=35 μm, g=25 μm, w=5 μm with vary distance *d* between CW and U-shape structure. The geometrical parameters of (**d**) are $P_{x}=P_{y}=120\;\;\mu$m, L=88 μm, l=29 μm, g=5 μm, w=5 μm with vary distance between CW and double-SRRs structure. The geometrical parameters of (**e**) are $P_{x}=P_{y}=120\;\;\mu$m, L=108 μm, R=20 μm, w=5 μm, $\alpha =20^{\circ }$, with vary distance *d* between CW and double-CSRRs structure.

**Figure 2:**
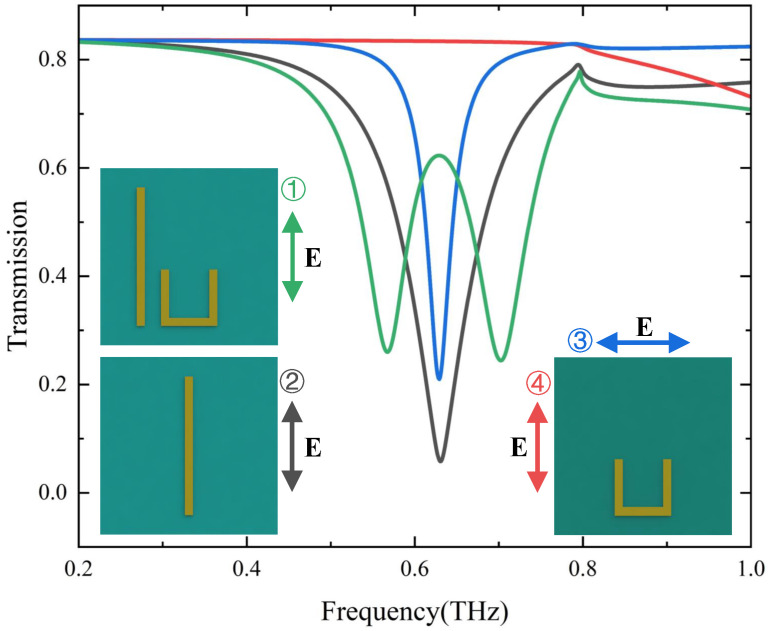
Transmission spectra of individual bright and dark modes structures and the whole structure. The black line represents the transmission spectrum of the single CW with *y* polarization (corresponding to the bright mode ➁). The blue and the red lines are the transmission spectra of the excited dark mode (single U-shape) with *x* polarization (➂) and unexcited dark mode with *y* polarization (➃), respectively. The green line represents the EIT transmission spectrum with the coupling of the bright and the dark modes (corresponding to the whole structure with *y* polarization ➀).

**Figure 3:**
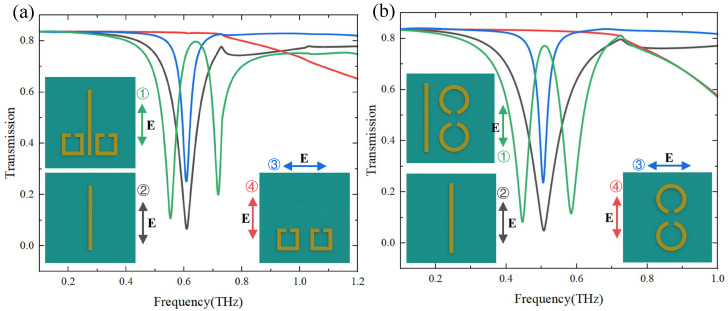
Transmission spectra of individual bright and dark modes structures and the whole structure. (**a**) The black line shows the transmission spectrum of a single CW with *y* polarization (corresponding to the bright mode ➁). The blue and the red lines are transmission spectra of the excited dark mode (two SRRs-shape) with *x* polarization (➂) and unexcited dark mode with *y* polarization (➃), respectively. The green line represents the EIT transmission spectrum with the coupling of the bright and the dark modes (corresponding to the whole structure with *y* polarization (➀). (**b**) The black line shows the transmission spectrum of a single CW with *y* polarization (corresponding to the bright mode ➁). The blue and the red lines are the transmission spectra of the excited dark mode (two CSRRs-shape) with *x* polarization (➂) and unexcited dark mode with *y* polarization (➃), respectively. The green line represents the EIT transmission spectrum with the coupling of the bright and the dark modes (corresponding to the whole structure with *y* polarization (➀).

As shown in Figure 1c–e, the unit cell contains bright modes structures of cut wires (CWs), and different dark modes structures (U shape, SRRs, CSRRs) can acquire EIT. The geometric parameters of the structure have been given in the graphic notes of Figure 1. We simulated three different EIT metasurface structures to obtain the bright mode and the dark mode at different polarizations and the corresponding EIT transmission spectra in Figure 2. and Figure 3. The numbers in different colors in the insets of Figure 2 and Figure 3 correspond to the transmission spectra of various structures under different polarization directions of the incident electromagnetic wave. Based on the substrate material and metal structure (CWs) of the single bright mode, we continuously varied the size of CWs to observe the resonant frequency shifts and changes in the transmission spectrum. We have selected the resonant frequency of the bright mode to be approximately 0.6 THz, while structures exhibiting other resonant frequencies can also be chosen. The incident electromagnetic wave is *y*-polarized at this time, while it becomes *x*-polarized when simulating the dark mode. Moreover, since changing the distance between the bright-mode and dark-mode structures can modulate their coupling strength, it is also necessary to choose an appropriate period of the unit cell structure. Transmission anomalies are observed at approximately 0.8THz in Figure 2 and 0.7 THz in Figure 3, which can be attributed to the lattice mode of the periodic structure. Lattice modes, also called Rayleigh anomalies, exist in the metasurface structure of periodic arrays. The frequency of lattice mode is defined as follows [18]

(14) $$f_{LM}^{\left[i,j\right]}=\frac{c\sqrt{i^{2}+j^{2}}}{P(\sqrt{\varepsilon }+\sin\theta )},$$

where *i* and *j* are a pair of integers that define lattice mode orders, *P* is the period of the structure, θ is the incident angle of terahertz waves, and ε is the relative permittivity of the substrate (11.7 in simulation). We only consider the case of normal incidence, so θ = 0. The frequency of the (0,1) order lattice mode is calculated to be 0.7906 THz when the period is 110 μm, corresponding to the transmission anomaly in Figure 2. The frequency of the (0,1) and the (1,1) order lattice modes are calculated to be 0.7247 THz and 1.0249 THz when the period is 120 μm, corresponding to the transmission anomalies in Figure 3. Higher order lattice modes beyond the frequency range of interest are not addressed in this discussion. The frequency of the lattice mode is closely related to the period of the unit cell structure and the relative permittivity of the material according to the definition of the lattice mode frequency. Therefore, when selecting the device period, it is important to minimize the impact of the lattice mode on the transmission spectrum as much as possible. Furthermore, during the same period, a metasurface structure of a single dark mode is simulated, and by adjusting the structural parameters, the resonance frequency of the dark mode under x-polarization approaches that of the bright mode under y-polarization.

Firstly, we take the CW as a bright mode structure and the U-shape as a dark mode structure in this example (Figure 2). We assume that the polarization direction of the excited THz wave is *y*, and the external electromagnetic wave can only directly excite the bright mode (CW) as shown in the black line of Figure 2 but not the dark mode (U-shape) as shown in the red line of Figure 2. The bright mode can excite the dark mode with an electromagnetic field due to the coupling between them. Thus, we simulate the transmission spectrum of the metasurface structure with only dark mode under *x* polarization for the same period as shown in the blue line of Figure 2. The bright and dark modes have similar resonance frequencies when excited by electromagnetic waves with different polarization directions. When the bright mode and dark mode are placed in the same unit cell, a high transmission transparent window is opened near the original low transmission resonance frequency due to the coupling effect between them, as shown in the green line of Figure 2.

Furthermore, we change the structures of dark modes to perform an EIT-like effect in metamaterial, as shown in Figure 3. We take CWs with different lengths as the bright modes and SRRs (CSRRs) as the dark modes. We demonstrate the transmission spectra of dark modes excited by *x* polarization incident wave to represent the dark modes excited by bright mode due to the coupling between two modes, as shown in blue lines in Figure 3a,b. When we introduce the bright mode structure into the unit cell, the coupling between bright modes and different dark modes can also acquire EIT-like phenomenons as shown in green lines in Figure 3.

The electromagnetically induced transparent metasurface is a typical example of bright mode and dark mode coupling. When we add a layer of metal on the back of the substrate to prevent the incident light from passing through, thus, the transmission metasurface converts to a typical metal-dielectric-metal absorber. In the theoretical section, we present the coupling equation of the bright mode and the dark mode and combine it with the interference theory. By solving the equations, we obtain the relationship between the absorption frequencies of the bimodal absorber and the resonance frequencies, losses of the bright mode and the dark mode, and the coupling strength between the bright mode and the dark mode structures (see Equation (13)). Considering the relationship between the basic physical parameters such as resonance frequency, loss, and coupling strength of the structure and the geometric parameters of the metasurface structure, the effect of these parameters on the spectral response can be analyzed according to the Equation (13).

**Figure 4:**
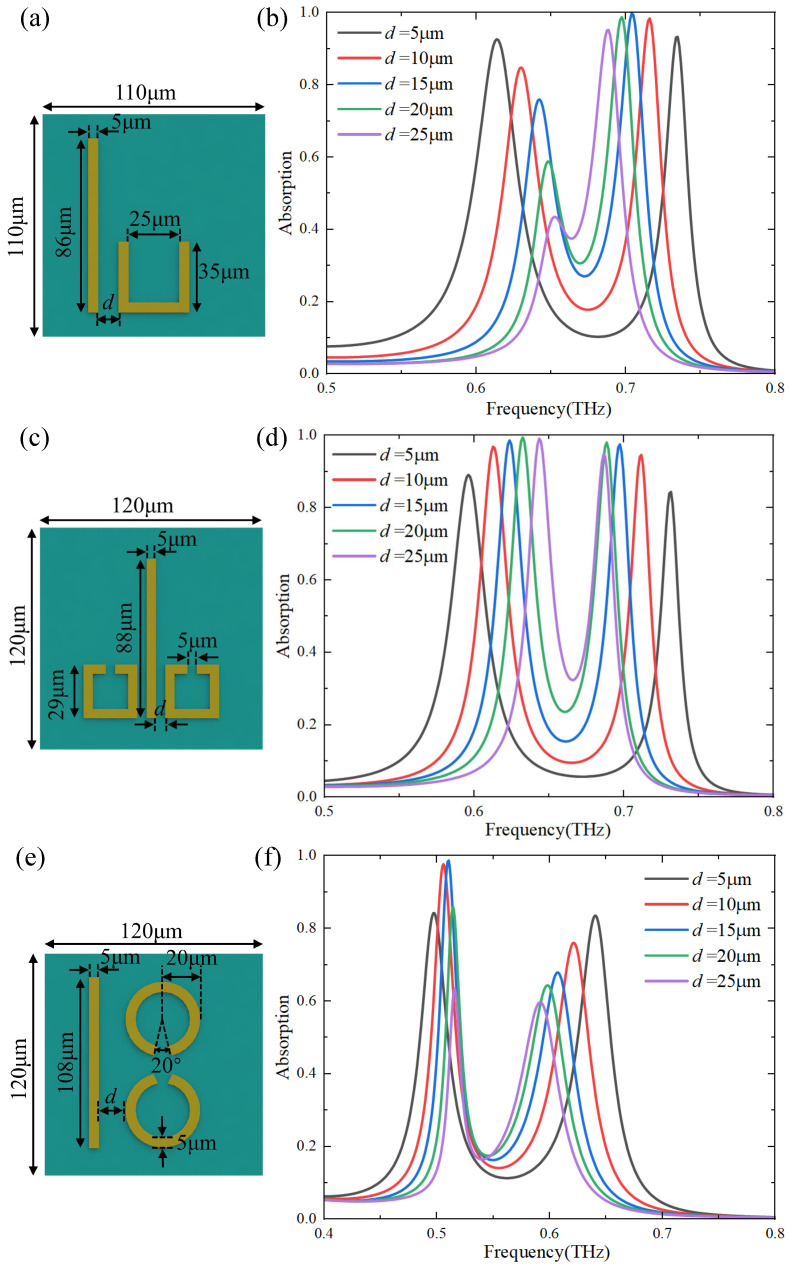
(**a**,**c**,**e**) are three typical EIT metasurface structures with different geometrical parameters of bright and dark modes. (**b**,**d**,**f**) are the corresponding absorption spectra with different distances *d* between the bright modes (CWs) and the dark modes (U-shape, SRRs-shape, and CRRs-shape) structures.

We demonstrate that the frequencies of absorber shifts as the distance between structures varies in a unit cell in Figure 4. The curves with different colors in Figure 4 represent the absorption spectra of the two structures in one unit cell at different distances. According to the coupled mode theory, the coupling strength between structures decays exponentially as the distance between structures increases. To demonstrate the variation of the absorption peak frequency with the coupling strength between structures in one unit cell, as shown in Figure 4, the distance *d* is continuously increasing from d=5μm to d=25μm at 5μm intervals with other geometrical parameters fixed. From the results in Figure 4b,d,f, we can see that when the distance between the structures increases, which means that the coupling strength between the two modes decreases, and the two absorption peaks gradually move closer to each other (from black lines to purple lines). We perform the simulations by employing three different unit cell structures and find that the frequencies of both absorption peaks are getting closer to each other as the distance increases.

According to Equation (13), when the coupling strength *g* becomes larger, $\omega_{+}$ increases while $\omega_{-}$ decreases, and the difference between the frequencies of the two absorption peaks becomes larger. Therefore, the frequency difference between the two absorption peaks gradually increases as the coupling strength between the bright and dark modes increases, which is consistent with our analytical solution.

The difference between the three structures is mainly due to the composition of the dark mode structure and the relative position of the bright mode. The motivations behind the selection of structures in this paper are as follows: Firstly, we have chosen three commonly used metasurface structures that exhibit electromagnetically induced transparency as representative examples of bright mode and dark mode coupling. Furthermore, the selection of the three different dark mode structures will cause different coupling processes between the bright and the dark modes, but due to variations in the coupling strength, a similar absorption peaks frequencies shift effect can still be produced. In addition, our findings are not dependent on specifically engineered structures. Therefore, we utilize several kinds of typical EIT metasurface structures to illustrate our theoretical findings. It is only necessary for the resonant frequencies of the bright-mode and dark-mode structures to be approximately equal under different incident electromagnetic wave polarization directions due to the theoretical approximation that $\omega_{1}$ ≈ $\omega_{2}$, with no specific requirement for the shape of the structures. Therefore, through our selection of these diverse metasurface structures for simulations, we have achieved consistent conclusions that an increase in coupling strength leads to a decrease in frequency difference between two absorption peaks, thus confirming the generality of our theoretical findings.

## 4. Disscussion

**Figure 5:**
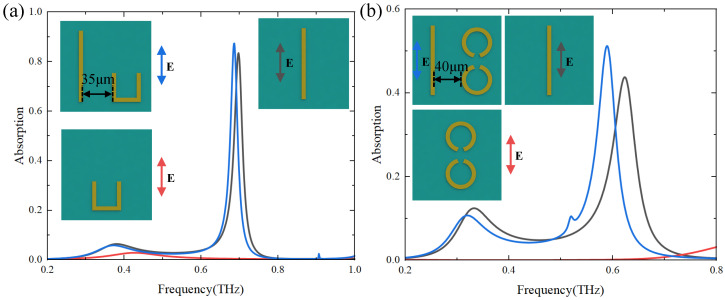
The absorption spectra of the whole structure (blue line) and the single bright mode (black line) and dark mode (red line) under *y* polarization with different structures (**a**) U-shape, and (**b**) CSRRs.

To further analyze the physical mechanism of the absorption peak variation, the distance between the bright and dark mode structures continued to increase in the simulation, as shown in Figure 4. According to the coupled-mode theory, when the distance between the bright mode and the dark mode increases, the coupling strength between them decreases, which leads to the weak excitation of the bright mode to the dark mode. As the distance increases, the absorption spectrum of the whole structure is more and more similar to the absorption spectrum when only the bright mode exists, and the absorption of the structure is mainly due to the contribution of the bright mode. When the coupling is further reduced, the dark mode will not be excited by the bright mode, and the two absorption peaks will become one absorption peak, and the frequency and absorption rate of the absorption peak are similar to that of the single bright mode, as shown in Figure 5a.

**Figure 6:**
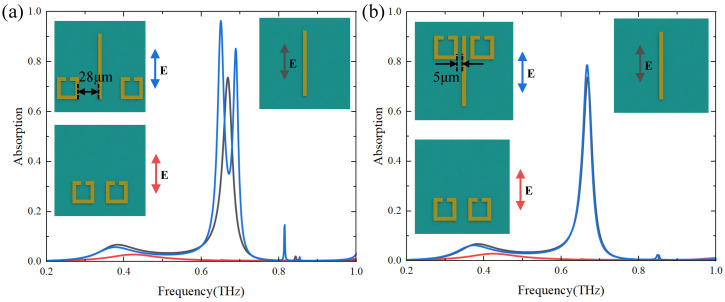
The absorption spectra of the whole structure (blue line) and the single bright mode (black line) and dark modes (red line) under *y* polarization with different positions (**a**) dy=−29.5μm, and (**b**) dy=29.5μm.

In order to demonstrate the universality of the results, we use two different metasurface structures with bright and dark mode coupling, as shown in Figure 5b and Figure 6. When the dark mode is two CSRRs located on the same side of the bright mode (CW), as shown in Figure 5b, as the distance between the two modes in a unit cell increases, the dark mode in the unit cell is closer to the bright mode in the next period. However, due to the strong coupling between the bright mode and the dark mode, the absorption spectrum of the whole structure is a little different from that of the single dark mode. The simulation results show that when the coupling between the two modes is further reduced, the absorption spectrum of the whole structure (blue lines) will be further similar to the absorption spectrum of a single bright mode (black lines). However, the absorption spectrum of the whole structures in Figure 6a is not as similar to the absorption spectrum of the single mode as in Figure 5a. Due to the limitation of the period, the distance between the bright and dark modes cannot increase to infinity, which means that the coupling between the structures will not disappear completely. As shown in Figure 6a, for structures whose dark modes are SRRs, when the distance between structures in a unit cell continues to increase, the distance of dark modes between adjacent structures will become smaller. However, due to the weak coupling between the dark modes of adjacent periods, it does not have an obvious effect on the spectrum of the whole structure. Although the two absorption peaks do not become one absorption peak, they still show a tendency to approach the absorption spectrum of the single bright mode.

When the period is increasing for simulation, according to the calculation formula of lattice mode [18], it can be seen that the lattice mode will move to low frequency with the increase of the period, which will affect the transmission spectrum. The anomalous transmission points in Figure 2 and Figure 3, all come from the influence of the lattice mode. In Figure 6b, without changing the period, the two SRRs are moved up along the direction of CW (y+ direction). Literature [28] has theoretically and experimentally verified that the coupling between the bright mode and the dark mode will decrease when the SRRs located on two sides of CW move along the direction of y+. dy represents the longitudinal offset between the geometric center of the SRR and the unit cell structure. At the position dy=29.5μm selected in Figure 6b, the coupling between the bright mode and the dark mode is weak that the bright mode will not be able to excite the dark mode at this time, and the absorption spectrum of the whole structure is very close to that of the single bright mode.

Therefore, the frequencies of the two absorption peaks generated by the coupling of the bright mode and the dark mode will move away from each other as the coupling strength increases, and approach each other as the coupling weakens. Under certain conditions, the two absorption peaks will merge into one, and the absorption spectrum of the whole structure will become more and more similar to that of the single bright mode, which is also consistent with our theoretical analysis. When the polarization direction of the incident electromagnetic wave is parallel to the single CW (*y*-polarization), the CW is excited, and the energy becomes localized on the single CW structure, resulting in very low transmittance, which shows a transmission valley in the spectrum, as shown in the black lines in Figure 2 and Figure 3. When the bright mode and the dark mode are coupled, the energy transfers from the bright mode to the dark mode, which causes the redistribution of the system energy, and a transparent window is opened around the resonance frequency of the bright mode, as shown in the green lines in Figure 2 and Figure 3. However, when the bright mode and dark mode are coupled, the energy of the bright mode is transferred to the dark mode, resulting in the generation of two absorption peaks by the dark mode. Furthermore, when the coupling between the two modes is weak, the absorption peak of the whole structure is close to the absorption peak when only bright modes are present, which is also proved by the results in Figure 5 and Figure 6b. At this stage, the absorption generated by the single dark mode under *y*-polarization is almost negligible, as indicated by the red lines in Figure 5 and Figure 6. Therefore, when the coupling between the structures is strong, the energy of the bright mode is transferred to the dark mode, and the absorption mainly arises from the dark mode. However, when the coupling between the structures is weak, it becomes challenging for energy exchange between the two modes, and as a result, absorption mainly originates from the bright mode.

**Figure 7:**
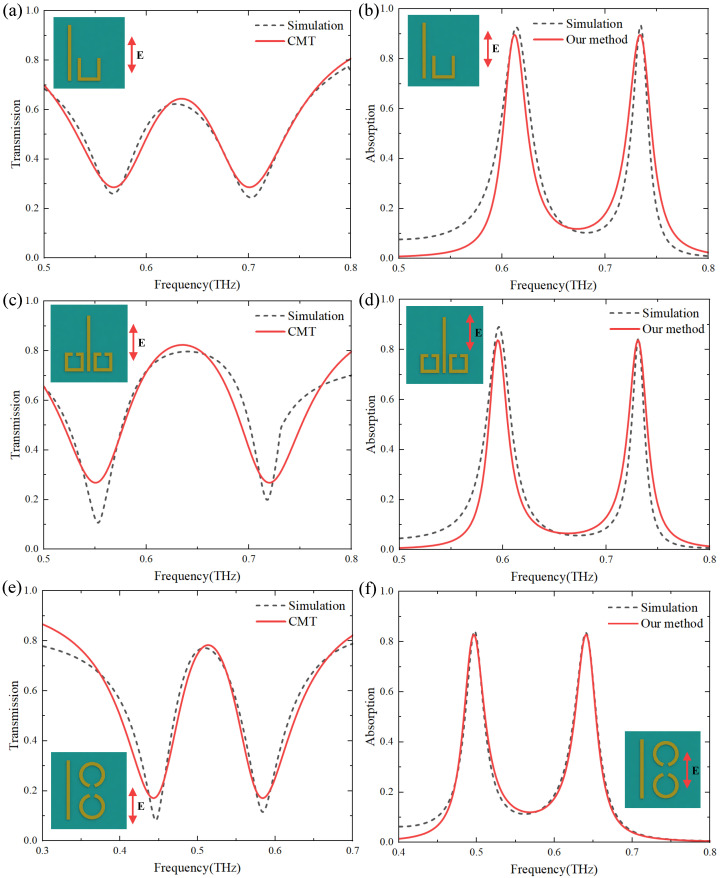
Simulation and theory spectra of three different structures, the transmission (**a**,**c**,**e**), and the absorption (**b**,**d**,**f**). The black dotted lines are the simulated results, and the red solid lines are the theoretical results.

To further verify the validity of the theory, as shown in Figure 7a,c,e, the transmission spectra of three different metasurface structures are calculated by employing CMT (Equation (8)), and the absorption spectra are calculated according to Equation (11) and $A=1-|r_{total}{|}^{2}$ [41]. The basic physical parameters required in the calculation are extracted from simulations of individual bright-mode and dark-mode structures. The theoretical and simulation results are in good agreement, except that the transmission spectrum shown in Figure 7c is affected by the lattice mode. Since the approximation that $\omega_{1}$≈$\omega_{2}$ is made during the derivation of the formula. Thus, the theoretical description is valid only if the resonant frequencies of the bright and dark modes are roughly the same.

**Figure 8:**
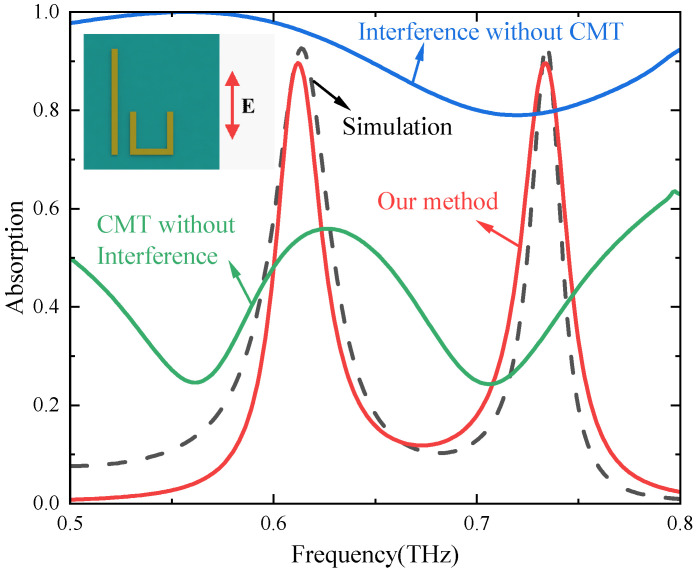
Simulation and theory spectra of the U-shape structure. The CST simulated result (black dotted line), the theoretical result obtained by our method (red solid line), the theoretical result by employing CMT without interference model (green solid line), and the theoretical result by interference model without CMT (blue solid line).

As illustrated in Figure 8, taking the U-shaped structure as an example, we employ various methods to calculate the absorption spectra of the metasurface structure. The black dotted line represents the results obtained by CST simulation, and the red solid line represents the theoretical results calculated by our proposed method, which are consistent with each other, further proving the effectiveness of the proposed method. The solid blue line in Figure 8 depicts the theoretical calculation result when only the interference model is used without considering coupling between two structures. The transmission and reflection coefficients are calculated according to Huygens’ principle without considering coupling [47]. The total reflection coefficient ($r_{total}$) is then calculated by combining the interference theory [41], and finally, the absorption spectrum is calculated according to the formula $A=1-|r_{total}{|}^{2}$ (solid blue line). The solid green line is obtained by using the transmission and reflection coefficients calculated by CMT combined with the calculation formula of the absorption spectra $A=1-{\left|R\right|}^{2}-{\left|T\right|}^{2}$ [41] without considering the effect of interference. The blue and green lines exhibit significant deviations from the simulation results, further confirming the validity of the theory proposed in the paper. Therefore, it is found that the results calculated by the proposed method are consistent with the simulation results and better than the results calculated by using only one theory.

Although the coupled-mode theory and interference theory pertain to distinct physical processes, their combination enables the establishment of a relationship between absorption frequency and fundamental physical parameters (such as resonance frequency, loss, and coupling strength) within the coupled-mode theory. The proposed method in this paper enables the inverse design of metasurface structure parameters for desired absorption frequencies, utilizing the close relationship between fundamental physical parameters in coupled-mode theory and geometric parameters of metasurface structures.

The inverse design of metamaterial devices can employ machine learning techniques which can also be applied to the inverse design of metamaterial absorbers. However, it is worth noting that machine learning methods [48,49,50,51,52] necessitate training on a substantial dataset acquired from simulations by scanning metamaterial geometry parameters. More importantly, deep learning methods do not incorporate the physical mechanism of optical phenomena. Furthermore, if the structure of the metamaterial is changed, the network needs to be retrained with a new dataset. However, this inverse design method obviates the need for extensive datasets and network training, thereby significantly reducing design time and overcoming the limitations of machine learning in addressing non-linear problems. Therefore, these problems can be avoided by using the analytical method proposed in this study for the inverse design. Nevertheless, the proposed method exhibits universality in coupling between bright and dark modes in metal metamaterials.

However, proposing a new method for the inverse design of absorbing metamaterials is just one aspect of the motivation in this study. Our primary focus is to investigate the impact of the coupling between the bright mode and dark mode on absorption frequencies. We present the analytical solution (Equation (13)) of the absorption frequency obtained through theoretical derivation and analyze the shift of the absorption frequencies by combining simulation results.

## 5. Conclusions

In this study, we investigate three distinct metasurface structures exhibiting electromagnetically induced transparency (EIT), wherein a metallic reflective layer is introduced on the backside of the substrate to prevent the transmission of incident electromagnetic waves, resulting in an absorption effect and enable the conversion of metasurface electromagnetic response from EIT to bimodal absorption. By employing coupled mode theory and interference theory, we analyze the relationship between the two absorption frequencies and resonant frequencies of bright and dark modes, as well as their respective losses and coupling strength. These physical parameters are closely related to the geometrical parameters of the structure. Consequently, by manipulating these geometrical parameters along with inter-structure coupling, it becomes possible to tune the absorption frequency, thereby offering a novel approach for inverse absorber design.

## Data Availability

The original contributions presented in the study are included in the article, further inquiries can be directed to the corresponding authors.
